# Non-invasive hemodynamic monitoring in trauma patients

**DOI:** 10.1186/s13017-015-0002-0

**Published:** 2015-03-08

**Authors:** Matthias Kuster, Aristomenis Exadaktylos, Beat Schnüriger

**Affiliations:** Department of Visceral and Transplant Surgery, Bern University Hospital, Bern, Switzerland; Department of Emergency Medicine, Bern University Hospital, Bern, Switzerland; Department of Visceral Surgery and Medicine, Bern University Hospital, Bern, Switzerland

**Keywords:** Initial care, Hypovolemic shock, Non-invasive hemodynamic monitoring, Trauma

## Abstract

**Background:**

The assessment of hemodynamic status is a crucial task in the initial evaluation of trauma patients. However, blood pressure and heart rate are often misleading, as multiple variables may impact these conventional parameters. More reliable methods such as pulmonary artery thermodilution for cardiac output measuring would be necessary, but its applicability in the Emergency Department is questionable due to their invasive nature. Non-invasive cardiac output monitoring devices may be a feasible alternative.

**Methods:**

A systematic literature review was conducted. Only studies that explicitly investigated non-invasive hemodynamic monitoring devices in trauma patients were considered.

**Results:**

A total of 7 studies were identified as suitable and were included into this review. These studies evaluated in a total of 1,197 trauma patients the accuracy of non-invasive hemodynamic monitoring devices by comparing measurements to pulmonary artery thermodilution, which is the gold standard for cardiac output measuring. The correlation coefficients r between the two methods ranged from 0.79 to 0.92. Bias and precision analysis ranged from -0.02 +/- 0.78 l/min/m^2^ to -0.14 +/- 0.73 l/min/m^2^. Additionally, data on practicality, limitations and clinical impact of the devices were collected.

**Conclusion:**

The accuracy of non-invasive cardiac output monitoring devices in trauma patients is broadly satisfactory. As the devices can be applied very early in the shock room or even preclinically, hemodynamic shock may be recognized much earlier and therapeutic interventions could be applied more rapidly and more adequately. The devices can be used in the daily routine of a busy ED, as they are non-invasive and easy to master.

## Introduction

When managing trauma patients, it is crucial to evaluate the hemodynamic status to exclude hemorrhage. During the initial assessment, blood pressure and heart rate are commonly used to estimate blood loss. However, these parameters may be altered due to pain, hypothermia, neurogenic or cardiogenic shock or other factors related to the patient or to the injury. Moreover, analgesic, sedative or relaxing drugs may interfere with these conventional vital signs, thus making their interpretation difficult.

Therefore, other diagnostic tools are required for hemorrhage detection. It has been shown that cardiac output is substantially different in hypotensive patients with or without blood loss. Low cardiac output then indicates blood loss, whereas normal or elevated cardiac output implies that blood loss is unlikely and that there may be other reasons for hypotension [1]. This is in accordance with many studies that have demonstrated that surviving patients exhibit significantly different hemodynamic patterns from non-survivors, and that these differences are already apparent in the Emergency Department (ED). For example, it has been repeatedly shown that cardiac index is higher in survivors than in non-survivors [2-8].

Pulmonary artery catheter thermodilution is considered to be the gold standard for cardiac output measurement [9]. Unfortunately, the invasive nature of this method means that it is not applicable during the initial phase in the ED [9,10]. Thus, thermodilution often cannot be used early in the evaluation of trauma patients.

A non-invasive device that permits advanced hemodynamic monitoring as soon as the patient arrives in the ED or even preclinically would be of great benefit in the assessment of the hemodynamic state. However, before such a new device is introduced into clinical routine, it needs to be assessed in controlled clinical trials.

The purpose of this review is to evaluate the accuracy and clinical applicability of non-invasive hemodynamic monitoring devices in the early assessment of trauma patients.

## Methods

### Search strategy

A systematic literature search was conducted using PubMed as its primary source. Studies from January 1966 to July 2014 were considered. Multiple searches were performed using the following keywords: non-invasive hemodynamic monitoring AND/OR non-invasive cardiac output monitoring AND/OR thoracic electrical bioimpedance AND/OR impedance cardiography AND/OR bioreactance AND/OR NICOM AND trauma. In PubMed, the ‘related articles’ algorithm was employed to identify additional articles. Moreover, bibliographies of original reports and reviews were screened for additional citations. Preliminary screening was performed utilizing titles and abstracts. The full-length articles of potentially appropriate studies were retrieved for further screening.

### Inclusion and exclusion criteria

**Figure 1:**
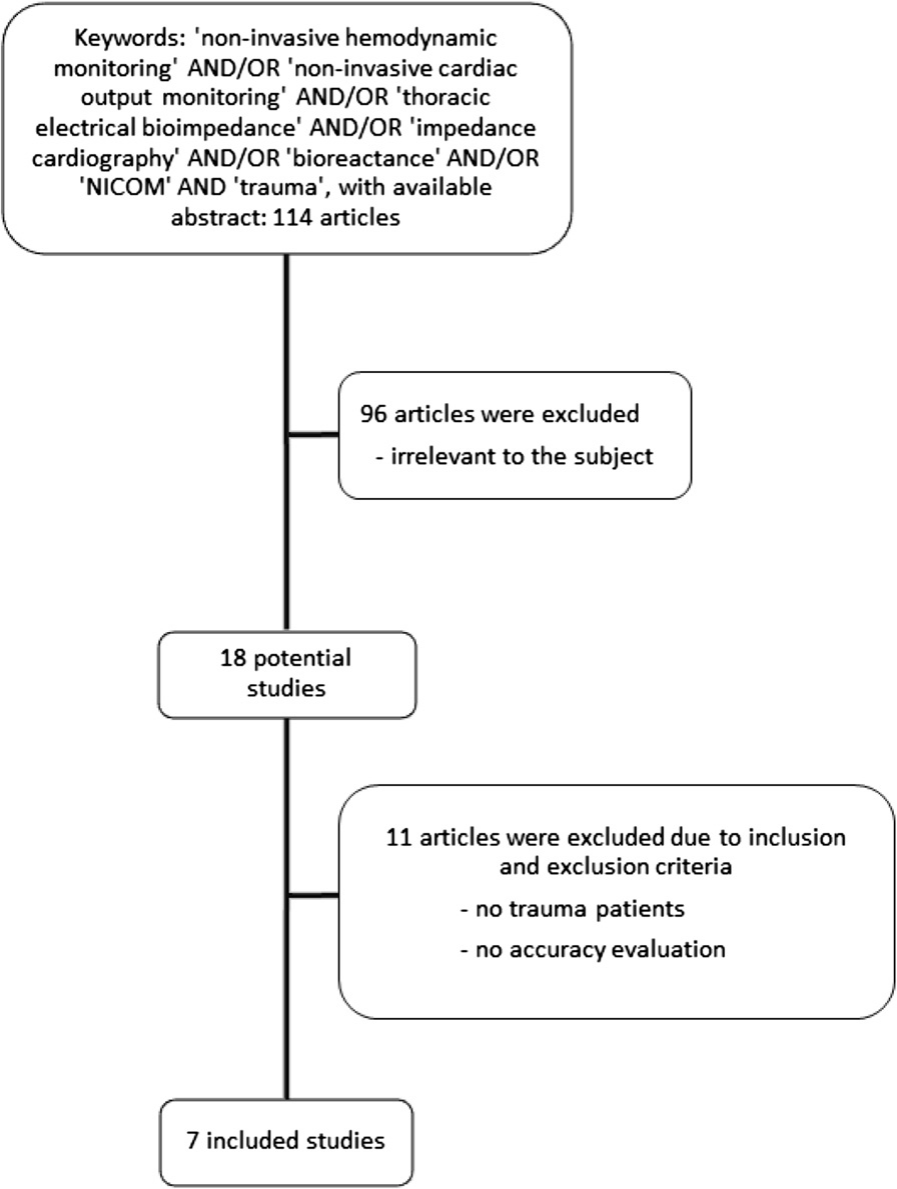
**Study selection process.**

Only those studies were considered for inclusion that explicitly investigated the accuracy of non-invasive hemodynamic monitoring devices in trauma patients by comparing it to the pulmonary artery catheter thermodilution method (Figure 1). Devices were examined that measure at least cardiac output through thoracic electrical bioimpedance, or through variations of this technology, such as bioreactance. Both prospective and retrospective studies were considered for inclusion. Studies in languages other than English, reviews, case reports or case series of <10 patients, were not considered for inclusion.

### Data extraction

Data was extracted on the accuracy of non-invasive hemodynamic monitoring devices. The devices measurements had to be compared to pulmonary artery catheter thermodilution, the gold standard for cardiac output measuring [9]. Correlation coefficients were taken from the articles, together with bias and precision analysis between the non-invasive hemodynamic monitoring device and pulmonary artery catheter thermodilution. If obtainable, the limits of agreement were also extracted.

Besides data on accuracy, attention was paid to the device’s usability in the ED, including advantages and limitations due to the device’s mode of operations and its mode of displaying data. Finally, the new devices’ possible clinical impact in the ED was considered.

### Characteristics of devices

For non-invasive hemodynamic monitoring, the most commonly used method is the thoracic electrical bioimpedance technology. Eleven non-invasive disposable prewired hydrogen electrodes need to be placed on predefined locations on the skin. Three electrodes function as electrocardiography leads and are placed across the precordium and left shoulder [2,4-8,11-13]. The other eight are positioned in pairs so that they lie over the top and bottom of the lung [5,11]. Each pair consists of an injecting and a sensing electrode. Two injecting electrodes are placed on the lateral side of the neck, opposing each other, and the other two on each side of the chest at the level of the xiphisternal joint. The injecting electrodes send a 100-kHz, 4-mA alternating current through the patient’s thorax, and the sensing electrodes measure voltage differences which change during the cardiac cycle. Each contraction of the hearth ejects the stroke volume into the aorta, which reduces the impedance (resistance) across the chest, as the electrical signals preferentially travel down the aorta, rather than passing through the aerated alveoli of the lung. Thus, an electrical pulsatile impedance curve is captured by the sensing electrodes. This curve is used to calculate the baseline impedance (Zo) and the first derivative of the impedance waveform (dZ/dt). The bioimpedance signals and the electrocardiogram are filtered with an all-integer-coefficient filtering technology to decrease processing time. The digital signal processing also uses time-frequency distributions to increase signal-to-noise ratios. Thus, the device is able to calculate stroke volume, which is multiplied by heart rate to get cardiac output [11].

The bioreactance method is a modification of the thoracic electrical bioimpedance technology [10]. It is based on an analysis of relative phase shifts of an oscillating current that occur when this current traverses the thoracic cavity. Four dual-electrode stickers need to be placed on the skin. Each sticker consists of an outer injecting electrode that emits a high-frequency sine wave into the body, and an inner receiver electrode that is used by the voltage input amplifier. Two stickers are placed left and right on the upper thorax, while the other two are placed on the lower thorax. The stickers on a given side of the body are paired, so that the currents are passed between the outer electrodes of the pair and voltages are recorded from the inner electrodes. The system detects the phase shift of the input signal relative to the injected signal. The change in the phase shift over time is correlated with the blood volume in the aorta, which fluctuates with the cardiac cycle. This allows the calculation of stroke volume [14].

## Results

### Study selection

Figure 1 shows the study selection process. A total of 114 studies were identified using the aforementioned search strategy. The abstracts were screened, which revealed 18 studies with the potential for inclusion. After obtaining the full-length articles, a total of 7 studies were included [2,3,5,6,11,13,15]. Their publication dates ranged from 1996 to 2006. All these studies compared the performance of a non-invasive hemodynamic monitoring device to the invasive pulmonary artery thermodilution method. They are summarized in Table 1.

**Table 1:** **Evaluation studies on the accuracy of thoracic electrical bioimpedance devices**

**Author, year**	**Study design**	**Device**	**Patients**	**Correlation coefficient r**	**r** ^**2**^	**Bias and precision (l/min/m** ^**2**^ **)**
Bishop et al. 1996 [15]	Prospective	Renaissance Technologies	54 patients with gunshot wounds	0.79	0.62	−0.011
Shoemaker et al. 1998 [11]	Retrospective	Renaissance Technologies	268 (139 trauma patients)	0.83	0.68	−0.058 +/- 0.78
Velmahos et al. 1999 [2]	Prospective	Renaissance Technologies	38 severely traumatized patients	0.91	0.83	-
Velmahos et al. 1999 [3]	Prospective	Renaissance Technologies	134 blunt trauma patients	0.83	0.69	−0.02 +/- 0.78
Shoemaker et al. 2001 [6]	Prospective	IQ System; Wantagh Inc.	151 trauma patients	0.91	0.83	−0.3 +/- 1.1
Brown et al. 2005 [13]	Retrospective	IQ System; Wantagh Inc.	285 critically injured patients	0.84	0.71	−0.14 +/-0.73
Shoemaker et al. 2006 [5]	Prospective	IQ Model 101; Noninvasive Medical Technologies LLC or PhysioFlow; VasoCOM	267 trauma patients	0.92	0.84	−0.07 +/- 0.47

The seven studies used devices that are based on thoracic electrical bioimpedance methodology [2,3,5,6,11,13,15]. These devices included a system from Renaissance Technologies, Newtown, Pennsylvania, the IQ System from Wantagh Inc., Bristol, Pennsylvania, the IQ Model 101 from Noninvasive Medical Technologies LLC, Las Vegas, Nevada, and the PhysioFlow from VasoCOM, Bristol, Pennsylvania.

### Accuracy of devices

Seven studies evaluated the accuracy of the cardiac output measurements by a thoracic electrical bioimpedance device and correlated this with the measurements of the invasive pulmonary artery catheter thermodilution method [2,3,5,6,11,13,15]. Table 1 gives an overview of the studies designs, the devices used and the data that was extracted.

All authors calculated the correlation coefficient, by comparing the cardiac output measured by the thoracic electrical bioimpedance device to the invasive thermodilution method. Moreover, all but one study conducted a bias and precision analysis [3,5,6,11,13,15]. The newest study also calculated the limits of agreement between the two methods [5].

Four studies published between 1996 and 1999 used a “new thoracic electrical bioimpedance device”, developed by Renaissance Technology, Newtown, Pennsylvania [2,3,11,15]. The first study was conducted by Bishop et al. in 1996 [15]. Here, patients with gunshot wounds were assessed. The correlation was r = 0.79, r^2^ = 0.62. Bias was -0.011 l/min/m^2^. Only a fraction of the cardiac output measurements were performed in the ED, and most came from the intensive care unit (ICU) [15].

In a study by Shoemaker et al. published in 1998, correlations between the pulmonary artery thermodilution method and the thoracic electrical bioimpedance device by Renaissance Technologies were calculated separately in the ED, the ICU, and the Operating Room (OR) [11]. However, this series did not consist of trauma patients only. 52% (139 of 268) of the subjects had trauma-related injuries, while the rest consisted of medical, non-trauma emergencies. The correlation coefficient for the entire population in the ED was r = 0.83, r^2^ = 0.68. Bias and precision were -0.058 +/- 0.78 l/min/m^2^. In the OR, these values improved to r = 0.88, r^2^ = 0.77, bias and precision = -0.027 +/-0.46 l/min/m^2^, but these differences were not statistically significant. The authors considered that the overall performance was satisfactory [11].

In 1999, Velmahos et al. evaluated 38 severely traumatized patients on arrival in the ICU with the thoracic electrical bioimpedance device from Renaissance Technologies, Newtown, Pennsylvania [2]. The investigators calculated a correlation coefficient of r = 0.91, r^2^ = 0.83, which they regarded as reasonably satisfactory. However, as the pulmonary artery thermodilution method was initiated after ICU arrival, these findings do not reflect the device’s performance in the ED [2]. A second study by the same author included 134 patients with blunt trauma who were assessed with the same devices on arrival in the ED. A correlation coefficient of r = 0.83, r^2^ = 0.69 was found. Bias and precision were -0.02 +/- 0.78 l/min/m^2^ [3].

The IQ System from Wantagh Inc., Bristol, Pennsylvania is also based on the electrical bioimpedance technology and was evaluated in two studies.[6,13] One was published in 2001 by Shoemaker et al. [6]. These investigators calculated a correlation coefficient of r = 0.91, r^2^ = 0.83. Bias and precision were -0.3 +/- 1.1 l/min/m^2^. The population in this study consisted of 151 trauma patients and the measurements were performed in the ED [6]. The second study using the IQ System was executed by Brown et al. in 2005 and included 285 critically injured patients, with either blunt (85%) or penetrating traumas (15%) [13]. In this study, the influence of the patient’s age on the performance of non-invasive cardiac output measurement was specifically evaluated. The investigators were concerned that atherosclerosis and a rigid thoracic aorta could falsify the results. The study population was stratified into three age groups: <55, 55-70, and >70 years old. The correlation coefficients were 0.82, 0.87, and 0.80, respectively, while bias and precision were -0.17 +/- 0.76 l/min/m^2^, -0.04 +/- 0.61 l/min/m^2^, and -0.04 +/-0.60 l/min/m^2^, respectively. Thus, good correlations between the cardiac output values of the IQ System and the pulmonary artery thermodilution method were found, and no statistically significant differences were detected between younger and older patients [13].

Shoemaker et al. published a study in 2006 which evaluated the IQ model 101 from Noninvasive Medical Technologies LLC, Las Vegas, Nevada, and the PhysioFlow from VasoCOM, Bristol, Pennsylvania [5]. Both devices are based on thoracic electrical bioimpedance. The correlation coefficient was r = 0.915, r^2^ = 0.84. Bias and precision was -0.07 +/- 0.47 l/min/m^2^. This was the only study to calculate the limits of agreement (accuracy) between the bioimpedance and thermodilution methods, which was 19.7% and considered to be acceptable [5]. It has been suggested that limits of agreement up to +/- 30% should be accepted when evaluating cardiac output monitoring devices, because pulmonary artery thermodilution itself has an inherent measurement error of 10 to 20% [16,17].

## Discussion

A total of five prospective observational and 2 retrospective studies investigating the accuracy of non-invasive hemodynamic monitoring devices in trauma patients are currently available. The thoracic electrical bioimpedance methodology was used in all of these studies. The accuracy of non-invasive cardiac output monitoring was broadly satisfactory.

### Practicability and limitations of devices

Accuracy remains the most important aspect when evaluating a new method or technology in patients’ hemodynamic monitoring. However, its limitations, usability and convenience in daily clinical routine are also important.

There are important limitations to non-invasive cardiac output measurement that have been identified in the past by several authors. Motion artifacts, restlessness, shivering, anxiety, hyperventilation and agitation can interfere with the measurements [5,6,11]. However, all these circumstances may also limit the accuracy of pulmonary artery thermodilution and most other hemodynamic monitoring techniques [8,11]. Faulty electrode placement can obviously prevent good monitoring [5,6].

Moreover, extensive pulmonary edema, pleural effusion, valvular heart disease, dysrhythmias, extensive chest wall edema, and chest tubes parallel to the aorta can reduce the impedance measured by the device, and lead to false data [5,6,8,11]. In this case, the device’s measurements do not provide a reliable basis for clinical decisions [11].

Besides the limitations, some authors have described specific advantages of the non-invasive hemodynamic monitoring devices. One is the continuous, on-line display of measurements [2,3,5,6,8,11,15,18]. This and the real-time data presentation are very convenient [3,5,8,18]. It allows instant recognition of circulatory deterioration and supports clinical decisions [2-4,11-13,15].

Another point that has been emphasized is that non-invasive devices can be applied very early in the ED [2-4,7,8,11,13,18]. The safety of the technology for both patients and staff has been emphasized by many authors [3,5,7,8,11,13,18]. The devices are very mobile and convenient, which allows their use at the bedside [7,8,11,18]. Their use is easy, quick and user-friendly [1,3,6,11,15]. Finally, feasibility is good, as the learning curve is short [3,5].

### Clinical impact

Most investigators have used the bioimpedance device with other non-invasive techniques, such as pulse oximetry, or measurements of transcutaneous oxygen and carbon dioxide tension and non-invasive blood pressure [2-8,11,12,18]. Thus, the clinician has indicators of cardiac function (cardiac output, stroke volume), pulmonary function (oxygen saturation) and tissue perfusion (oxygen and carbon dioxide tension) [2,4].

The aforementioned early applicability of non-invasive monitoring devices may solve a key problem of invasive hemodynamic monitoring. It is known that invasive hemodynamic techniques have important limitations, especially in the treatment of trauma patients. For example, inserting pulmonary artery catheters is time-consuming, susceptible to complications, and personnel-intensive, and may be difficult in severely injured patients [1-3]. Moreover, these catheters require a sterile critical care environment and the cessation of other — possibly more urgent — interventions [2-5,7]. In contrast, non-invasive methods may be applied very early in the initial evaluation of the trauma patient, even preclinically [2,11]. Moreover, they do not interfere with clinical management [3]. The continuous real-time display of measurements permits early recognition of circulatory abnormalities or deterioration, which makes it possible to perform early therapeutic interventions and to recognize their hemodynamic effects [2,11]. Shoemaker et al. concluded that non-invasive monitoring is of great value as a “front end” device and may bridge the time to invasive monitoring [11]. Moreover, the physiological parameters measured by the device may permit early recognition of shock and hypotension [3,5,6]. Earlier therapeutic intervention could then be facilitated when time is crucial [2,6]. Furthermore, non-invasive monitoring can be used to titrate therapy to appropriate therapeutic goals [5].

Dunham et al. conducted a study on 270 consecutive trauma activation patients in which they evaluated a non-invasive cardiac output monitoring device [1]. These investigators concluded that the multiple associations of cardiac output with patient conditions imply that non-invasive hemodynamic monitoring provides an objective and clinically valid, relevant, and discriminate measure of cardiac function in acutely injured trauma activation patients. Moreover, they stated, that the use of non-invasive hemodynamic monitoring may be associated with a shorter length of stay in surviving patients with complex injuries [1].

Shoemaker et al. considered that non-invasive monitoring could provide a means to develop an organized coherent therapeutic plan based on physiological criteria measured in the ED. This plan would accompany the patient as he/she proceeds to the OR, the radiology department or the ICU [6].

### Future outlook

New devices with potentially better accuracy are emerging. These devices should be evaluated for their impact in routine work when taking care of traumatized patients.

Early differential diagnosis of hypotension in the initial evaluation of trauma patient might be an important advantage of non-invasive hemodynamic monitoring. Knowing the patient’s cardiac index early may help the physician to differentiate between blood loss and other causes of hypotension [1]. Thus, these devices may help in determining the etiology of the illness.

If more were known about the hemodynamic changes in bleeding trauma patients, this would help the clinician in using the information gained by non-invasive devices. Therefore, more data is required to interpret the measurements performed early in the ED, e.g. for estimating blood loss.

## Conclusion

The accuracy of non-invasive cardiac output monitoring devices in trauma patients is broadly satisfactory. As the devices can be applied very early in the shock room or even preclinically, hemodynamic shock may be recognized much earlier and therapeutic interventions could be applied more rapidly and more adequately. The devices can be used in the daily routine of a busy ED, as they are non-invasive and easy to master. However, the impact of non-invasive cardiac output monitoring on patients’ outcome is uncertain and more clinical experience is warranted.
